# Cross-sectional associations of subjective sleep quality, chronotype and social jetlag with perceived stress in a sample of Irish adults

**DOI:** 10.1007/s41105-026-00640-0

**Published:** 2026-03-04

**Authors:** Rachael M. Kelly, Andrew N. Coogan

**Affiliations:** Department of Psychology, Maynooth University, National University of Ireland, Maynooth, Co. Kildare Ireland

**Keywords:** Stress, Sleep, Social jetlag, Chronotype, PSQI

## Abstract

To examine the relationships between subjective sleep quality, chronotype and social jetlag with perceived psychological stress in a sample of Irish adults. An observational cross-sectional study of 400 adults. Subjective sleep quality was assessed with the Pittsburgh Sleep Quality Index, chronotype and social jetlag were assessed with the Munich Chronotype Questionnaire, and psychological stress was measured with the Perceived Stress Scale. Correlational, groupwise and path analyses were applied to the data to examine the relationships between perceived stress and sleep variables, age and sex. Bivariate correlation analyses revealed statistically significant associations between both social jetlag and mid-sleep on free days and perceived stress (small effects), and moderate associations between subjective sleep quality and perceived stress. Groupwise analysis revealed that individuals in the high perceived stress group displayed greater social jetlag and poorer sleep quality, but no difference in chronotype, when compared to those with low or moderate stress. Path analysis revealed a moderate reciprocal relationship between subjective sleep quality and perceived stress, no direct effects of chronotype or social jetlag on perceived stress and a small indirect effect of average nightly sleep duration on perceived stress mediated through subjective sleep quality. Chronotype and social jetlag have minimal relationships with perceived stress, whilst subjective sleep quality has a moderate reciprocal relationship with perceived stress.

**Supplementary Information:**

The online version contains supplementary material available at 10.1007/s41105-026-00640-0.

## Introduction

Stress occurs when an individual does not have adequate resources relative to the demands of a given situation [1]. Stress may have environmental, psychological, or biological bases and generally causes activation of the sympathetic nervous system and the hypothalamic pituitary adrenal (HPA) axis [2]. Stress is broadly understood to be adaptive over short periods of time, but prolonged stress has been shown to have negative impacts on mental and physical health [1, 2]. Importantly, many of the components of the physiological stress system, such as HPA and sympathetic autonomic activity, are also under the control of the circadian clock, an intrinsic biological timekeeping system that produces daily rhythms in many physiological, endocrine, cognitive and behavioural outcomes [3]. Further, the circadian clock is a key shaper of sleep/wake behaviour and timing, and sleep duration and quality appear to have bidirectional associations with both acute and chronic stress; for example stress-related rumination may contribute to maladaptive cognitions underpinning insomnia disorder [4]. Conversely, improving sleep quality through cognitive behavioural therapy can increase resilience to stress [5] and sleep quality influences acute stress reactivity [6].

In terms of perceived psychological stress and sleep, inter-relationships between stress perception and sleep health parameters have previously been described. For example, a study during the COVID-19 pandemic reported a relationship between greater perceived stress and poorer subjective sleep quality [7]. In young adults, academic and emotional stress has been associated with poor subjective sleep quality [8]. Poorer subjective sleep quality has been proposed to be a mediator between high perceived stress and physical health outcomes such as cardiovascular disease [9]. Circadian characteristics of sleep timing have also been linked with psychological stress: later chronotype/eveningness has been associated with increased perceived stress [10]. Individuals with later chronotype are also more likely to experience high levels of social jetlag, a mismatch between internal biological time and social schedules characterised by differences in sleep timing on “work” and “free” days [11, 12]. SJL has previously been described to associate with stress responses in university workers [13]. A recent study on acute psychomotor stress and sleep quality and timing demonstrated small to moderate associations between acute stress and sleep quality, sleep duration, social jetlag, but not chronotype, suggesting that social jetlag associations with stress might be independent of chronotype [14].

In order to further investigate associations between stress and sleep timing and quality in adults, we examined the nature of these associations to determine the inter-dependency of sleep timing and quality factors’ association with perceptions of daily stresses. Specifically, we sought to describe associations between chronotype, social jetlag, sleep duration and subjective sleep quality with perceived stress and to use path analysis to assess whether associations of these sleep factors with perceived stress were direct or mediated through other factors in a sample of adults.

## Materials and methods

### Participants

Convenience sampling through social media, flyers and posts online was used to recruit participants. Some students were also recruited through the Department of Psychology’s research participant pool, made up of all full-time psychology students from Maynooth University in their second year of study. Participants had to be at least 18 years old to partake in the study and to have clearly demarcated “work” and “free” days as part of their usual weekly schedule. Due to the COVID-19 pandemic no responses were collected between March and September 2020 to control for the changing nature of the work environment and the impact of the national lockdowns; after this period had passed and individuals had acclimatised to the new schedule, recruitment recommenced and concluded October 2020. 468 individuals completed an online questionnaire; of these responses, 17 participants were excluded due to work schedules at night, 49 further responses were excluded due to either alarm clock use on free days or stating no free days, two further participants were excluded due to unrealistic values being provided. The final sample with complete data was *N* = 400; 57% (*N* = 228) of the final sample data was collected before March 2020, and forty-three% of the sample data (*N* = 172) was collected in September/October 2020. This study was approved by the Biological Research Ethics Sub-Committee at Maynooth University (BSRESC-2019-009) and all research was conducted in accordance with the Declaration of Helsinki. Informed consent was obtained from all participants in order to proceed to the surveys.

All participants reported their age (in years), gender, current job-status (employed/unemployed/retired/student) and whether they worked full-time or part-time. Commute time to work and normal work start and end times were reported. Participants selected an occupation from a drop-down menu guided by the International Standard Classification of Occupations (ISCO, 2008). Participants were also presented with an open-ended box to describe their job so that classifications could be checked by the researcher. This classification system was then used to group participants as full-time workers, students, or non-traditional workers where they may not have had regular hours (i.e., homemaker, carers).

### Measures and procedure

The *Munich Chronotype Questionnaire* (MCTQ) was used to estimate participants’ underlying circadian phase of entrainment and SJL [15, 16]. This instrument asks about typical sleep behaviour on both workdays and free days over the previous month and has two sections with illustrative diagrams. The key variables of interest derived from MCTQ scores were average sleep duration across the week (SDweek), sleep-corrected mid sleep on free days (MSFsc, an indicator of chronotype), and social jetlag (SJL; the difference between the timing of midsleep on “work” days and “free days” [12, 16]). SJL was calculated as both a relative (with directionality as to whether free day sleep timing was earlier or later than workdays) and absolute values. The *Pittsburgh Sleep Quality Index* (PSQI; [17]), was used to evaluate subjective sleep quality over the previous month. The main output of the PSQI is a total score indicating subjective sleep quality, with higher scores indicating poorer sleep quality. The *Perceived Stress Scale* (PSS) measures perception of stress by assessing individual’s feelings and thoughts over the previous month [18]. The PSS-10 has 10 items that are all rated on a 5-point Likert scale from 0 (never) to 4 (very often). Items 4,5,7,8 were positively worded and were reversed before the total score was calculated, and total scores can range from 0 to 40 with higher scores indicate higher psychological stress [19].

When participants opened the survey link they were presented with detailed information on the study covering the purpose of the study, confidentiality, and data handling policies. Anyone under the age of 18 was automatically directed to the end of the survey. After consenting to proceed, participants submitted demographic details, information on work schedule and occupation before proceeding to complete the MCTQ, the PSQI and the PSS. All of these questionnaires had to be completed in one session and the participants required around 15 min to complete the surveys. The online Qualtrics instrument only saved responses that were 100% complete. Data collected and used in this study, as well as the STROBE checklist for observational cross-sectional studies and results of statistical analyses, are available at https://osf.io/ba8gf/.

### Data analysis

For descriptive data analyses, the distribution of all variables was visually inspected and Kolmogorov Smirnov tests were used to assess normality of distribution. For inferential analysis, one-way ANOVAs, ANCOVAs or Kruskal Wallis tests were used to examine differences between groups (student, employed, career/homemaker) for demographic, sleep and circadian variables depending on normality of the dependent variable distribution. Following AN(C)OVA testing, for statistically significant results post-hoc testing was applied with Bonferroni tests. When assessing relationships between variables Pearson’s r was used for normally distributed variables while Spearman’s rho was used when the data was non-normally distributed. As the approach of the study was exploratory and not hypothesis testing, adjustments for multiple hypothesis testing were not applied, and *P* < 0.05 was taken as indicating statistically significant differences. Following correlation analysis False Discovery Rate (FDR) analysis was applied by the Benjamin-Hochberg method with alpha set to 0.05 to determine whether statistically significant results persisted after control for FDR in the 21 associations examined. All analyses were conducted on SPSS version 26. JASP version 0.14.1.0. To inform the study sample size, an online statistics calculator (https://www.danielsoper.com/statcalc/default.aspx) was used to guide the minimum sample size. In order to detect a small effect size (0.02) of SJL as a predictor on PSS scores with an alpha level of 0.05 and a power of 0.8. in a presumptive regression analysis, a suggested minimum sample size was 390.This approach was taken on the a-priori assumption that SJL would have a weaker association with PSS than other factors such as PSQI score, and as such if the study were powered sufficiently to detect associations of SJL with PSS, then it would also be powered to detect other associations of interest.

Path Analysis was conducted in SPSS AMOS (IBM Corporation) to test conceptually driven direct and indirect paths onto PSS from the endogenous variables that were identified in univariate analysis as statistically significantly associating with PSS. Maximum likelihood estimation was used in the model, and since there were no missing data points no imputation was not required. Bootstrapping with 1,000 iterations was applied to allow for calculation of bias-corrected 95% confidence intervals for direct and indirect effects. Model fit indices were interpreted as per Schermelleh-Engel et al. [20]. The pathways specified in the model one were: (1) age influences MSFsc (as described widely [12]); (2) MSFsc influences SJL [21]; (3) both MSFsc and SJL have direct effects on PSS [14]; (4) sleep quality, assessed by the PSQI, has a direct effect on PSS [8] and conversely PSS scores exert effects on PSQI scores [22]; (5) average nightly sleep duration, as determined from the MCTQ, will exert an effect on PSQI score [17] and (6) age and sex have direct effects on PSS [23]. As the model specified is non-recursive due to the reciprocal relationship between PSQI and PSS, the error terms for PSQI and PSS were specified to co-vary. The overall aim of the path analysis was to investigate the inter-dependencies of MSFsc, SJL, sleep duration and sleep quality on PSS, and not to best account for variance in PSS; as such, we were primarily interested in describing the direct and indirect paths of interest rather than optimising model fit or maximising the variance in the stress measures accounted for.

## Results

There were 400 complete responses in this study; 80% respondents were female and the mean age was 33.63 years (SD = 12.80; Table 1). Respondents were students (either undergraduate or postgraduate; 22.5%), workers (74%), and individuals not in conventional 9 − 5 employment, usually caring for a child or elderly relative (3.5%). Of those employed, the majority identified as professionals (58.4%), followed by those working in sales and services (16.9%), administration (12.2%), assistant professionals (8.1%) and those who engaged in manual labour (i.e., farming, construction; 4.4%). Median relative SJL was 1:05 h (IQR = 1:12 h, range − 1:32 h to + 4:30 h) with 4.5% showing negative relative SJL; median absolute SJL was 1:08 h (IQR = 1:10 h; Table 1). 47.3% of participants displayed absolute SJL of less than one hour, 38.5% displayed SJL of 1–2 h and 14.2% displayed more than 2 h of SJL. The mean MSFsc of the sample was 04:18 (SD = 01:11) and ranged between 00:29 and 08:00. Participants slept an average 7:42 h per night (SD = 1:04 h). Median PSQI was 6.00 (IQR = 5.00, range = 0–17), with 48.8% of the sample being classified as poor sleepers (PSQI score of 6 or greater). Students were significantly younger, had greater SJL, later MSFsc, shorter sleep duration and higher PSQI scores than those in employment (Supplemental Table 1). A comparison of scores collected before and after the onset of the Covid-19 pandemic revealed no statistically significant differences on any of the measures examined (Supplementary Table 2).

**Table 1:** Descriptive statistics of the survey sample

Variable	Mean (SD)/*N* (%)
Sample size (N)	400
Age (years)	33.63 (12.80)
Sex	
Male	80 (20%)
Female	320 (80%)
Work Group	
Students	90 (22.5%)
Workers	296 (74%)
Non-traditional workers	14 (3.5%)
Worker sub-divisions	
Professionals	173 (58.4%)
Sales/Services	50 (16.9%)
Administration	36 (12.2%)
Assistant professionals	24 (8.1%)
Manual labour	13 (4.4%)
Sleep and Stress Scores	
SDw^$^	7:27 h (1:25)
SDf^$^	8:25 h (1:30)
SDweek	7:42 h (1:04)
MSFsc	4:18 h (1:11)
Absolute SJL^$^	1:08 h (1:10)
Relative SJL^$^	1:05 h (1:12)
PSQI^$^	6.9 (5)
PSS^$^	18.06 (6.8)

^$^ indicates variables that were not normally distributed and as such are reported as median (IQR)

### Associations of perceived stress with social jetlag sleep scores

Perceived stress was weakly positively associated with relative SJL (rho=0.127, *P*=0.011) and absolute SJL (rho=0.126, *P*=0.012; Supplementary Fig. 1). MSFsc was also weakly positively associated with perceived stress (rho=0.182, *P*<0.001). PSS was moderately positively correlated with PSQI scores (rho=0.54, *P*< 0.001). Sleep duration across the full week or on work days only did not associate with PSS scores (Table 2). All of the associations reported in Table 2 that were statistically significant remained so after adjustment for FDR for the 21 correlations reported.

**Table 2:** Correlations between the sleep and circadian measures and PSS in the full sample, students only and those in employment only

	Relative SJL	Absolute SJL	MSFsc	SDw	SDweek	PSS	PSQI
Relative SJL	1						
Absolute SJL	0.984***	1					
MSFsc	0.515***	0.491***	1				
SDw	− 0.019	− 0.036	0.113*	**1**			
SDweek	0.132**	0.118*	0.153**	0.906***	1		
PSS	0.127*	0.126*	0.182***	− 0.075	− 0.056	1	
PSQI	0.046	0.058	0.082	− 0.323***	− 0.316***	0.543***	1

Bold values indicate statistically significant correlations

*SDw* sleep duration on work days, *SDweek* average sleep duration across the week

*Indicates *P* < 0.05, **P < 0.01, and ***P < 0.001

**Fig. 1:**
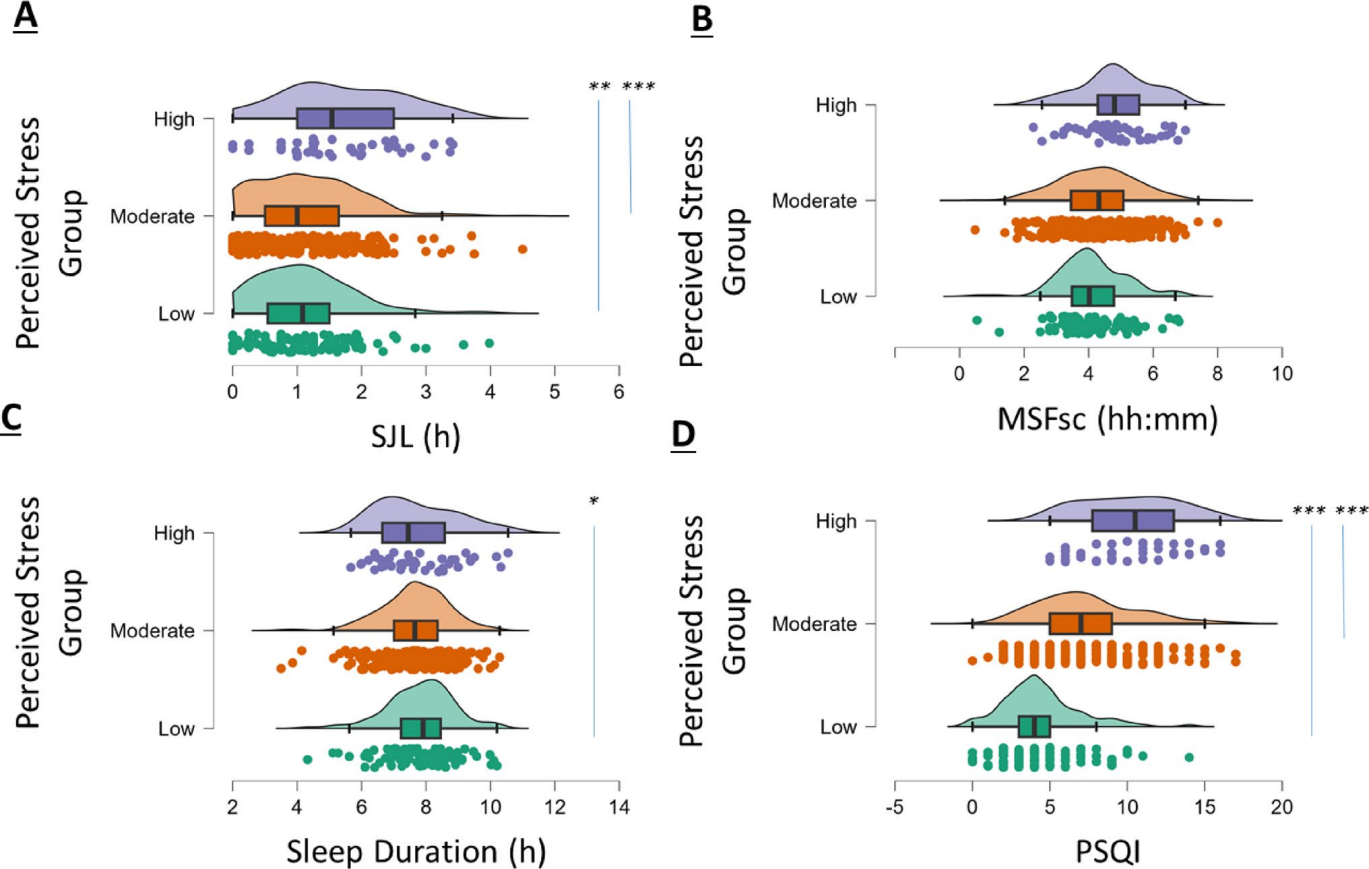
Raincloud plots showing groupwise distributions of **A** absolute SJL, **B** MSFsc, **C** average weekly sleep duration and **D** PSQI scores according to “low”, “moderate” and “high” perceived stress ratings. * indicates *P* < 0.05, ***P < 0.001 on

We next examined participants categorised as low, medium and high stress according to their scores on the PSS. As greater PSS and SJL were associated with age, we examined the association of PSS grouping (“low” PSS score up to 13, “moderate” PSS group from 14 to 26, “high” PSS score 27 or above) on sleep and circadian scores in a series of ANCOVAs controlling for age. There was a significant effect of PSS grouping on absolute SJL (F(2,395) = 7.64, *P* < 0.001), with higher PSS grouping associated with greater SJL on post-hoc testing (Fig. 1A). PSS group was not associated with differences in MSFsc (F(2,396) = 2.97, *P* = 0.053; Fig. 1B) but PSS grouping was associated with average sleep duration (F(2,396) = 3.83, *P* = 0.022; Fig. 1C) and greater PSS was associated with higher PSQI scores (F(2,396) = 60.05, P = < 0.001; Fig. 1D).

**Fig. 2:**
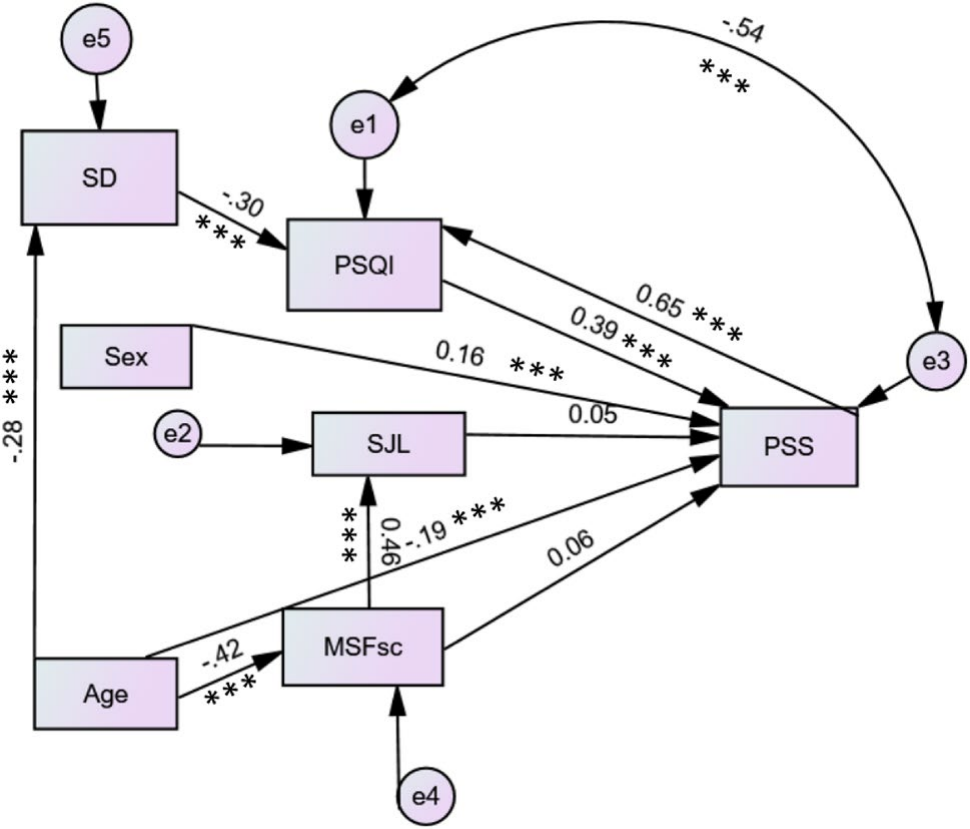
Path analysis of variables that had been shown in univariate analysis to have associations. E1-5 are latent variables representing the error terms of the endogenous variables in the model. Co-efficients indicated on paths are standardised. *** indicates *P* < 0.001. E-1–5 indicate the error terms for the endogenous variables in the model.* SD* average sleep duration across week (from MCTQ),* PSQI* total PSQI score,* PSS* total PSS score,* SJL* social jetlag,* MSFsc* – midsleep on free days, sleep corrected

To explore the potential directional relationships between PSS and sleep measures identified as being associated with PSS in univariate analysis, we undertook a path analysis. Age and sex were included as exogenous variables in the model as these factors are well understood to impact on sleep and stress. There were 28 distinct sample moments in the model and 18 parameters estimated, and as such there were 10 degrees of freedom. The Chi Square for the model was 7.201 (*P* = 0.723; CMIN = 0.720), and as such the model appeared to be sufficiently overspecified. The model fit indices were adequate (RMSEA = 0.00 (90% CI 0.00. 0.041), PCLOSONE = 0.981, GFI = 0.995, AGFI = 0.986, FMIN = 0.003, AIC of 43 compared to 56 for the saturated model and 482 for the independence model). The path analysis indicated significant small effects of sex and age onto PSS, but no statistically significant paths from MSFsc or SJL on PSS. PSS had a direct path onto PSQI, and reciprocally PSQI had a direct path onto PSQI (Fig. 2). Average sleep duration had a direct path onto PSQI. Analysis of the bootstrapped estimates for the direct and indirect effects on interest reveals that age and sleep duration had indirect paths onto PSS mediated through PSQI, and age and sex had direct paths onto PSS; neither MSFsc nor SJL had direct or indirect paths onto PSS (Table 3).

**Table 3:** Direct and indirect effects of the path analysis for sleep variables effects on PSS. Standardised (β) and unstandardized (b) are both presented

Path	β (95% CI)	b (95% CI)	*P*
Direct Effects			
Age-> MSFsc	−0.424 (−0.498, −0.347)	−0.039 (−0.048, −0.031)	< 0.001
Age-> SD	−0.284 (−0.379, −0.182)	−0.024 (−0.032, −0.015)	< 0.001
Age-> PSS	−0.187 (−0.272, −0.102)	−0.100 (−0.146, −0.055)	< 0.001
SD-> PSQI	−0.302 (−0.387, −0.214)	−0.987 (−1.276, −0.687)	< 0.001
Sex-> PSS	0.164 (0.090, 0.251)	2.799 (1.51, 4.31)	< 0.001
PSQI-> PSS	0.389 (0.176, 0.596)	0.759 (−0.335, 1.189)	< 0.001
MSFsc-> PSS	0.057 (−0.021, 0.143)	0.332 (−0.126, 0.849)	0.173
MSFsc-> SJL	0.464 (0.377, 0.542)	0.324 (0.251, 0.399)	< 0.001
SJL-> PSS	0.052 (−0.034, 0.143)	0.430 (−0.288, 1.209)	0.182
PSS-> PSQI	0.646 (0.439, 0.864)	0.331 (0.225, 0.451)	< 0.001
Indirect Effects			
MSFsc-> PSQI	0.051 (0.005, 0.102)	0.297 (0.028, 0.611)	0.030
Age-> PSS	−0.064 (−0.117, − 0.0.018)	−0.034 (−0.064, −0.010)	0.003
SD-> PSS	−0.157 (−0.252,−0.063)	−1.000 (−1.654,−0.392)	0.004

## Discussion

The current study examined the associations between subjective sleep quality and habitual sleep timing and duration with perceived stress in a cross-sectional sample of Irish adults. The main finding is that MSFsc or SJL do not have direct paths onto PSS, but that PSS has a moderate strength reciprocal relationship with PSQI. Sleep duration has an indirect path onto PSS through PSQI, and MSFsc has an indirect path onto PSQI presumably through PSS. These results that indicate that subjective sleep quality is the sleep factor of greatest importance regarding the relationship with perceived psychological stress, and that chronotype and SJL appear to have minimal associations with psychological stress measured by the PSS. Previous reports have indicated potential bidirectional relationships from bivariate analyses between stress and sleep quality, such that poorer subjective sleep quality associates with higher perceived stress [7, 24] and acute psychomotor stress [14]. Chen et al. [25] report that MSFsc and PSS both associate with PSQI scores in a small sample of Taiwanese students. Huang et al. [22] have reported that PSS associated with PSQI scores, and that this path from PSS to PSQI was largely mediated by anxiety and depression symptoms. Previous work on this relationship has reported that academic and emotional stress is self-reported as a cause of impaired sleep quality [8]. Resilience may mediate the effect of perceived stress on sleep quality [25], as may rumination and/or social anxiety [26]. Du and colleagues [27] report that in an international cohort, sleep quality mediates the relationship between perceived stress and eating behaviours and alcohol use, indicating that sleep quality might be a node for intervention to lessen unhealthy behaviours linked to chronic psychological stress. Poorer subjective sleep quality may impact on chronic psychological stress by increasing acute stress reactivity [5]; such effects would adversely impact on response to the daily stressors that the PSS assesses.

Previous work has shown that sleep quality has only small associations with chronotype and SJL [28]. In addition to PSQI we examined the association of MSFsc and SJL with PSS as such effects may be independent of relationships between PSQI and PSS scores; we report no direct paths of either MSFsc or SJL onto PSS. A study during the Covid-19 pandemic which noted marked decreases in SJL and changes in sleep timing reported that such reductions were not associated with changes in PSS [29]. Jokubauskas et al. [30] report no association between SJL or chronotype and perceived stress in students. SJL has been associated with the stress-related construct of anxiety: a recent study in South Korea demonstrates a that every hour increase in SJL is associated with a 35% increase in anxiety symptom rating [31]. Another study has reported that SJL associates with anxiety symptoms in a manner that is independent of sleep duration [32]. Further, greater SJL has been associated with greater likelihood of experiencing stress-related depressive symptoms [33]. Our current findings, showing lack of robust associations of sleep timing factors of MSFsc and SJL with PSS, indicate that associations between the sleep timing and stress-related constructs of anxiety and depression may be independent of stress; future studies might usefully address such issues.

SJL was present in the expected magnitude and prevalence in the study sample, with 47.3% of the sample displaying less than one-hour SJL, 38.5% of the sample displaying 1–2 h SJL and 14.2% of the sample displaying more than 2 h SJL. These levels are similar to those reported in a recent Japanese study investigating SJL and stress reactions [13]. Students in our sample had greater SJL than workers, likely due to their younger age associating with later MSFsc which in turn is associated with greater SJL [12]. As such, the lack of direct associations between SJL and PSS scores observed are unlikely to be due to an unusual distribution of SJL in the current sample. Differing accounts for the association of SJL with stress may be related to the psychometric or other measures used to assess differing stress constructs [34] and cultural factors relating to PSS measurements in different populations [35].

### Strengths and limitations

The current study has some important strengths. The MCTQ, PSQI and PSS are very well validated scales for assessing sleep timing and chronotype, subjective sleep quality, and perceived stress, and allowed for the examination of a number of different sleep dimensions for their relationship with perceived stress. The application of path analysis allowed us to examine putative directional relationships between sleep factors and perceived stress. There are also a number of important limitations. Firstly, sleep measures were only collected as self-report, and no objective sleep data was collected (for example, through actigraphy). Secondly, as noted previously, the relationships between stress and sleep may be mediated by, and impact on, other related cognitive and mental health factors such as rumination, anxiety and depression [25], but we did not examine such factors in the current study. Thirdly, the current study was cross-sectional in nature; as such the temporal and causal nature of the associations examined cannot be ascertained (e.g. does prior and/or current perceived stress impair subjective sleep quality?); future work utilising longitudinal study designs might usefully address such questions. Given the cross-sectional nature of the data, the reciprocal paths between PSS and PSQI described here should be treated as evidence for the theoretical plausibility of such relationships but not treated as empirical evidence for the existence of such; longitudinal study design and data would be required to confirm and better describe such relationships. Next, there was a female preponderance in the study sample, with 80% of participants being female, and as such findings may not generalise to other populations. Finally, the COVID-19 pandemic which started mid data collection. To account for potential implications of this, no data was collected from March 2020 until September 2020, and 43% of the sample was recruited from September – December 2020, a period during which there were still changes in people’s lives and schedules which altered sleep factors. To determine the effect that this had on our primary variables we compared the group that were recruited before and after the onset of the COVID-19 pandemic for our primary variables; SJL, MSFsc, sleep duration or PSS did not differ statistically significantly between the two recruitment windows of the study.

## Conclusion

The current results reinforce the concept that subjective sleep quality might be an important point of intervention to improve psychological stress, and vice versa in the alternate direction. However, the current results also indicate that chronotype and social jetlag may not be effective targets thought which to improve psychological stress in adults. Further work with study designs allowing for direct causal analysis of the relationships between sleep factors and perceived stress incorporating more possible mediating and moderating factors such as measures of affect, temperament and rumination would build on the current findings and address some of key weaknesses of the present study.

## Supplementary Information

Below is the link to the electronic supplementary material.

Supplementary Material 1
